# Computational Structural Analysis Predicts Host-Range Promiscuity and Antiviral Resistance in North American H5N1 Lineages

**DOI:** 10.34133/csbj.0066

**Published:** 2026-05-08

**Authors:** Sayal Guirales-Medrano, Kary Ocaña, Khaled Obeid, Rachel Alexander, Colby T. Ford, Daniel Janies

**Affiliations:** ^1^ Center for Computational Intelligence to Predict Health and Environmental Risks (CIPHER), University of North Carolina at Charlotte, Charlotte, NC, USA.; ^2^ Department of Bioinformatics and Genomics, University of North Carolina at Charlotte, Charlotte, NC, USA.; ^3^ National Laboratory of Scientific Computing, Rio de Janeiro, Brazil.; ^4^ Department of Biological Sciences, University of North Carolina at Charlotte, Charlotte, NC, USA.; ^5^ School of Data Science, University of North Carolina at Charlotte, Charlotte, NC, USA.; ^6^ Tuple LLC, Charlotte, NC, USA.

## Abstract

Influenza A virus has been circulating in birds in Eurasia for more than 146 years, but human infection has been sporadic. H5N1 (clade 2.3.4.4b) has recently infected hundreds of species of wild and domestic birds and mammals in North America. Infections include 71 people in the United States. There have been 2 human fatalities (United States and Mexico). We have integrated time-series analysis, molecular phylogenetics, and structural biology to understand how H5N1 is circulating in North America and adapting to new hosts. Our time-series analysis reveals that the circulation of H5N1 follows a distinct seasonal pattern, with cases in the United States increasing November to April. We also document an increase in the number of cases reported since 2021. We show that H5N1 spreads in North America as 2 distinct lineages. These viral lineages have achieved a vast host range by efficiently binding the viral surface protein hemagglutinin to both mammalian and avian cell surface receptors. This novel host-range promiscuity is concomitant with the strengthening of the viral polymerase basic 2 protein binding for mammalian and avian immune proteins. Once bound, the immune proteins have diminished ability to fight the virus, thus allowing for efficient replication. Our analyses predict that while most antivirals remain effective, a fatal human isolate showed reduced binding to multiple drugs from different classes. The H5N1 virus is causing an animal pandemic through promiscuity of host range and strengthening ability to evade the innate immune systems of both mammalian and avian cells.

## Graphical Abstract

**Figure:**
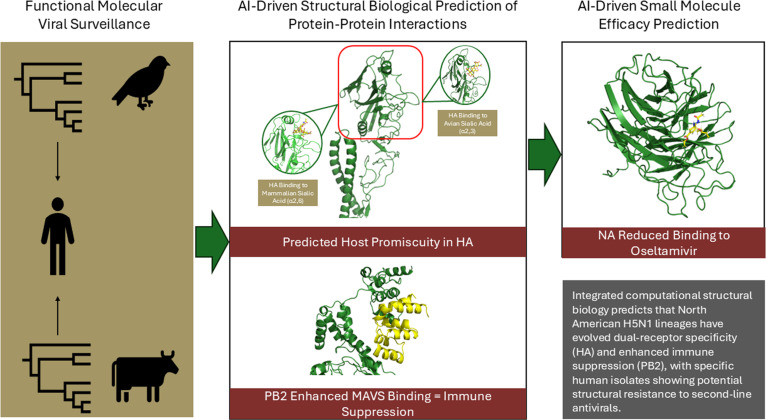

## Introduction

The history of human civilization is marked by recurrent influenza pandemics, which have been exclusively caused by influenza A viruses (IAVs) originating from avian or swine reservoirs, primarily maintained by Anseriformes (ducks and geese) and Charadriiformes (gulls and shorebirds) as the vast natural reservoir for nearly all IAV subtypes [1]. While typically asymptomatic in natural hosts, zoonotic spillover can be catastrophic, as evidenced by the 1918 H1N1 “Spanish Flu,” which resulted in an estimated 50 million human deaths worldwide [2]. Subsequent pandemics in 1957, 1968, and 2009 highlight the perpetual threat [3,4] driven by antigenic drift and shift [5].

Among the various avian influenza subtypes, H5N1 has garnered substantial global concern. A highly pathogenic avian influenza (HPAI) H5N1 virus first detected in 1996 in farmed geese in Guangdong, China, belonging to the Goose/Guangdong (Gs/GD) lineage [6]. This lineage demonstrated zoonotic potential in Hong Kong in 1997 [7] and has since evolved and spread across Asia, Europe, Africa, and, more recently, the Americas [8]. The virus has also been responsible for more than 985 human infections with a case fatality at almost 50%, although sustained human-to-human transmission has not been established [9]. We provide a complete account of the history of H5N1’s geographic spread in our recent paper [10]. While avian lineages have long circulated in Eurasia [11], H5N1 clade 2.3.4.4b was detected in wild birds of the United States in December 2021 [12]. It has since spread across the continent (Fig. S30), infecting more than 246 species [13], including mammals and agricultural animals (File S1) [14,15].

As of February 2025, the United States has confirmed 71 human cases [16], including a fatality linked to genotype D1.1 [17], and suffered massive poultry losses [18]. Canada reported similar widespread infection (File S2) [19,20]. In Mexico, while reports focused primarily on wild and agricultural birds [21,22], a fatal pediatric case (genotype D1.1) occurred in April 2025 [13,23].

IAVs are members of the Orthomyxoviridae family, characterized by a genome of 8 single-stranded, negative-sense RNA segments. The critical barrier to human adaptation for avian influenza viruses is the receptor specificity of the hemagglutinin (HA) protein. Avian viruses typically bind α2,3-linked sialic acids found in the bird enteric tract, while human viruses bind α2,6-linked sialic acids dominant in the human upper respiratory tract [24]. A switch in this specificity is considered a prerequisite for human pandemic potential [7]. While HA governs entry into the host cell, the magnitude of the species barrier is equally determined by the viral polymerase complex, specifically the polymerase basic 2 (PB2) subunit [25]. PB2 is the primary driver of viral replication. Even if HA allows entry to the host cell, the virus cannot achieve productive infection in mammals unless PB2 adapts to the mammalian nuclear environment and suppresses innate immune signaling [26].

Antiviral medications are cornerstones of clinical management and pandemic preparedness for influenza [27]. Two primary classes of drugs are licensed in most countries that target the proteins neuraminidase (NA) and polymerase acidic (PA). NA inhibitors, such as oseltamivir and zanamivir, prevent viral egress from infected cells, and polymerase inhibitors, like baloxavir and marboxil, targets the PA subunit to block viral gene transcription [27]. However, the high mutation rate of IAVs presents a constant threat to the efficacy of these drugs. Amino acid substitutions within the viral target proteins can confer resistance, diminishing therapeutic options. For H5N1, which is known to evolve rapidly, it is crucial to monitor mutations in NA, PA, and other polymerase components to ensure that these vital medical countermeasures remain effective [28].

In this study, we developed a computational structural biology workflow, supported by phylogenetic techniques, to investigate how currently circulating H5N1 (clade 2.3.4.4b) strains are overcoming the species barrier in North America. We combined genomic and syndromic surveillance data with high-resolution protein modeling and molecular docking to simulate the physical interactions between evolving viral proteins and host factors. We assess adaptation using computational analyses of natural selection of the nucleotide diversity of the hemagglutinin (HA), neuraminidase (NA), matrix protein (MP), polymerase acidic (PA), polymerase basic 1 (PB1), and polymerase basic 2 (PB2) genes.

We hypothesized that the unprecedented spread of H5N1 among birds and mammals (wild and domestic) and humans is driven by specific structural adaptations that allow H5N1 to enter mammalian cells without losing avian receptor affinity and moreover replicate well in mammals and birds. This viral trait is termed “host promiscuity” [29,30].

We investigated how mutations in HA interact with various host-cell receptors and how mutations in PB2 in the replication complex allow blocking of the hosts’ innate immune system by interacting with the mitochondrial antiviral signaling (MAVS) protein, human Importin-α3 human Acidic Nuclear Phosphoprotein 32 Family Member A (ANP32A) and avian orthologs. Finally, given the reliance on antivirals for pandemic mitigation, we modeled the binding affinity of current small-molecule drugs against these emerging strains.

## Methods

### Data acquisition and preparation

The H5N1 HA dataset from Ford et al. [10] was updated with sequences released in the latter half of 2024 and early 2025. We sourced the data from the Global Initiative on Sharing All Influenza Data (GISAID; gisaid.org) EpiFlu nucleotide sequence database and the United States National Institutes of Health’s GenBank (National Center for Biotechnology Information, NCBI; ncbi.nlm.nih.gov) database [31–33]. The initial dataset comprised 18,289 HA sequences. Additionally, sequences for PB2 (*n* = 3,415 sequences), MP (*n* = 29,011), NA (*n* = 18,466), PA (*n* = 17,873), and PB1 (*n* = 14,089) were collected. Sequences were aligned using MAFFT and manually curated to remove incomplete reading frames and trimming terminal unaligned regions (“ragged edges”) [34].

### Phylogenetic and evolutionary analyses

For all datasets, stop codons were trimmed using MACSE (v2) and manual review to prepare for selection analyses [35]. Phylogenetic trees for all genes were inferred using RAxML (v8.2.12; [36]) under GTRGAMMA. Outgroups were selected for each gene tree: LC718258 (A/jungle crow/Hokkaido/0104B085/2022) for HA, EPI3509783 (A/chicken/Montana/24-001136-002-original/2024) for PB2, EPI460427 (A/turkey/Ontario/6213/1966) for NA, NC_00736 (Goose/Guangdong/1996) for MP, GU052523 (A/chicken/Scotland/1959) for PA, and GU052524 (A/chicken/Scotland/1959) for PB1. Candidate vaccine sequences (e.g., GISAID: EPI1846961 for HA; GenBank: OQ958046 for NA, OQ958047 for MP, OQ958043 for PA, OQ958058 for PB1, and OQ958041 for PB2) were added as reference baselines. Analyses of natural selection were performed using the HyPhy package (v2.5.67; [37]). Selection pressure was evaluated using the Nielsen and Yang [38] suite of codon models (M0, M1a, M2a, M7, and M8). These models were implemented within the HyPhy software package by executing its internal positive selection modules through custom control scripts. This approach allowed for the estimation of the ratio of nonsynonymous to synonymous substitution rates (dN/dS) per codon within the HyPhy framework while adhering to the classic model definitions and likelihood-ratio tests typically associated with phylogenetic analysis by maximum likelihood. Amino acid sites being under positive selection were determined with a dN/dS >1.

### Epidemiological surveillance

**Fig. 1.**
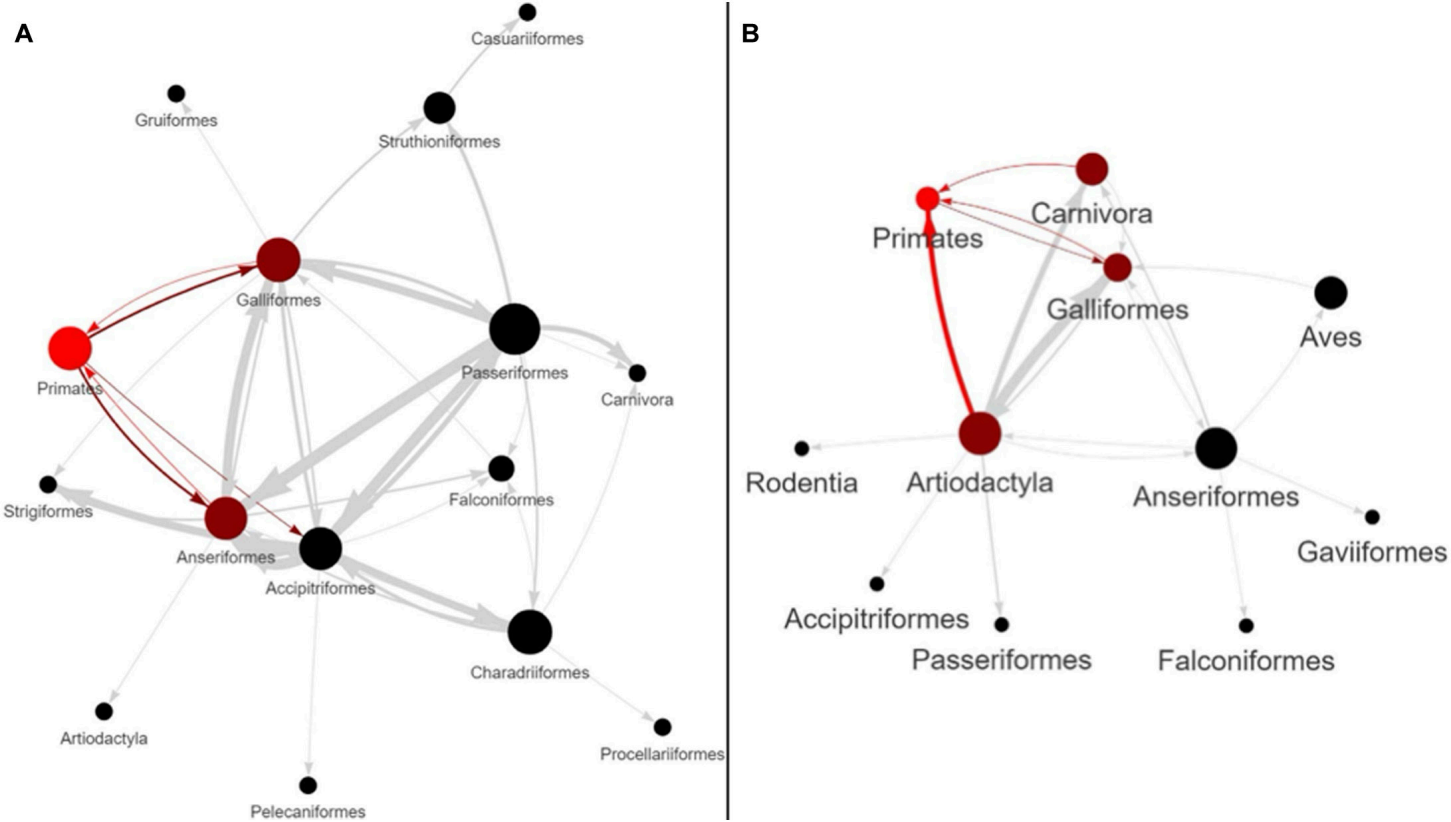
StrainHub networks created from subclades of the hemagglutinin (HA) sequence dataset using a betweenness-centrality metric. The arrows represent the directionality of viral transmission events recovered. The thickness of the lines represents a higher frequency of transmission. (A) Clade of interest surrounding the deceased patient infected with H5N1 in Louisiana in 2024 (PQ809550). The colors represent highlighted zoonotic events such as bidirectional transmission between Primates (humans), Galliformes (e.g., poultry), and Anseriformes (e.g., ducks and geese), and unidirectional transmission from Primates to Accipitriformes (e.g., hawks and eagles). (B) Clade of interest surrounding the human-bovine-avian transmission events in 2024. The colors represent highlighted zoonotic events such as unidirectional transmission from Artiodactyla (e.g., cows) to Primates (humans), bidirectional transmission between Primates and Galliformes (e.g., poultry), and unidirectional transmission from Carnivores (e.g. domestic cats) and Primates.

From our collected sequence data and corresponding metadata data, we created 2 additional visualizations for epidemiological trends related to H5N1. Using StrainHub, transmission networks were then generated, from the phylogenetic trees, for HA subclades to visualize transmission dynamics between host metadata (Fig. 1). We used the betweenness-centrality metric to determine these networks in StrainHub [39].

A time-series case dataset was generated from the GISAID EpiFlu database. Filters for subtypes H5 and N1 were applied, in addition to location, which was filtered to be exclusively within the United States. The data were then tabulated based on the collection date of each viral isolate. Data were similarly collected from the United States Department of Agriculture (USDA) Animal and Plant Health Inspection Service (APHIS). Data were aggregated from 3 databases, including the “HPAI Confirmed Detections for Backyard Poultry,” “Detections of Highly Pathogenic Avian Influenza in Mammals,” and “Detections of Highly Pathogenic Avian Influenza in Wild Birds.” Each APHIS data source was filtered for all cases collected from 2021 December 1 to 2026 March 18. Additionally, data were included from the NCBI GenBank Repository. All data were sourced from the NCBI Virus platform. Filters were applied for “Virus/Taxonomy,” “Genotype,” “Geographic Region,” and “Collection Date.” The following search terms were used for each filter, respectively: “Influenza A virus, taxid:11320,” “H5N1,” “USA,” and “from December 1, 2021 to March 18, 2026.”

These data were also analyzed through a seasonal decomposition method. Using the seasonal_decompose() method from the statsmodels Python library, a decomposition of the time series using moving averages resulted in separate time series that described the trend, the seasonality, and the residual (error) of the original data [40].

### Structural biology and molecular docking

#### HA structural analysis

Three-dimensional structures were generated for 4 HA protein sequences of interest: GISAID: EPI3171488 and EPI1846961 (vaccine candidate); GenBank: PQ591824 and PQ809550. Amino acid sequences were folded using ColabFold (v1.5.2; [41]). To simulate interactions with host receptors, human and avian sialic acid (SA) glycan structures were obtained from Protein Data Bank (PDB) entries 4K63 and 4KDO, respectively. Protein–glycan docking was performed for each HA model against both human and avian SA glycans using HADDOCK3 (v2024.12.0b7; [42]) to assess potential differences in binding affinity. HADDOCK3 docking parameters followed the default protein–glycan configuration file (https://github.com/haddocking/haddock3/blob/main/examples/docking-protein-glycan/docking-protein-glycan-full.cfg).

#### PB2 structural analysis

A similar structural analysis was conducted for the polymerase basic 2 (PB2) protein. Sequences from strains OQ958041 (vaccine candidate), PQ809562, PQ591825, and PP577947 were selected. Additionally, sequences carrying mutations at position 588 [GISAID: EPI3304108 (A588T), EPI3315580 (A588S), and EPI3447228 (A588V)] were included.

Known host interaction partners of PB2 were selected as docking targets: human MAVS (PDB: 2MS8), human importin-α3 (PDB: 4UAE), its avian ortholog (UniProt: A0A2I0M1B5), human ANP32A (PDB: 6XZQ), and its avian ortholog (NCBI: XP_075287452). Each protein target selected showcases a different interaction with influenza’s PB2. MAVS is responsible for innate immune response signaling of Janus kinase 1, which is inhibited by PB2 interactions [43]. Importin-α3 promotes transport of proteins into the host nucleus, i.e., importing PB2 into the nucleus for viral replication [25]. Lastly, ANP32A was selected due to its ability to mediate and increase viral replication when interacting with PB2 [44]. All protein structures were folded using ColabFold. Protein–protein docking simulations between PB2 variants and host targets were conducted using HADDOCK3 to evaluate binding interactions. HADDOCK3 docking parameters followed the default protein–protein configuration file (https://github.com/haddocking/haddock3/blob/main/examples/docking-protein-protein/docking-protein-protein-full.cfg).

#### Antiviral docking analysis

To investigate potential variations in antiviral susceptibility, a structural docking analysis was performed on key H5N1 protein targets. The selected proteins included NA, a surface glycoprotein that mediates viral egress, and the 3 components of the RNA-dependent RNA polymerase complex: PA, PB1, and PB2. Sequences for these proteins were obtained from NCBI and GISAID for a panel of representative strains:

• A/chicken/Scotland/1959 (historical reference)
• A/American Wigeon/South Carolina/22-000345-001/2021 (vaccine candidate)
• A/Astrakhan/3212/2020 (vaccine candidate)
• A/cattle/Texas/24-009110-004/2024 (bovine-to-human transmission)
• A/Texas/37/2024 (bovine-to-human transmission)
• A/California/150/2024 (bovine-to-human transmission)
• A/Louisiana/12/2024 (fatal avian-to-human transmission)

The corresponding antiviral compounds were selected based on their known protein targets: oseltamivir and zanamivir (NA inhibitors), baloxavir (PA inhibitor), favipiravir (PB1 inhibitor), and pimodivir (PB2 inhibitor). The Simplified Molecular-Input Line-Entry System (SMILES) strings for each antiviral were retrieved from the PubChem database [45].

The computational workflow for this analysis was as follows: The 3D structure of each protein variant was predicted using ColabFold. Concurrently, the SMILES strings for the antiviral compounds were converted into 3D molecular structures using RDKit [46]. Putative binding pockets on each target protein were identified using P2Rank [47]. Finally, protein–ligand docking simulations were conducted between each antiviral and its respective target protein using HADDOCK3 to evaluate their binding interactions. HADDOCK3 docking parameters followed the default protein–ligand configuration file (https://github.com/haddocking/haddock3/blob/main/examples/docking-protein-ligand/docking-protein-ligand-full.cfg).

#### Validation of structural analyses

To validate the predictive accuracy and sensitivity of our computational docking pipeline, we performed a retrospective benchmarking analysis using historical influenza A variants with well-documented phenotypic shifts from Carrasco et al. [48]. Binding affinities were primarily evaluated using Van der Waals’ (VDW) interaction energies. To assess host-receptor predictive accuracy, we modeled the historical H5N6 A/Black Swan/Akita/1/16 HA protein (PDB: 9NR2) as wild type and the same HA with a Q226L mutation (PDB: 9NR5). These HA proteins were docked against both avian (_α_2,3-linked; derived from PDB: 4K63) and mammalian (_α_2,6-linked; derived from PDB: 4K67) SA analogues.

Additionally, we validated the pipeline’s capacity to detect antiviral resistance by docking the oseltamivir against the wild-type NA (PDB: 2HU4) and its resistant counterpart harboring the classic H274Y mutation (PDB: 3CL0). Oseltamivir is part of the standard of care for influenza [49] (supplemental data: https://github.com/colbyford/Influenza_A_2.3.4.4b_Analyses/tree/main/Benchmark).

## Results

### Phylogenetic visualization analyses of host shifts

To understand the patterns of host transmission, we analyzed host category (avian and mammalian taxonomic orders) using StrainHub based on phylogenetic trees of HA nucleotide sequences.

This analysis revealed multiple independent zoonotic events, which we describe in detail below.

Based on these patterns, we identified 2 key subclades for in-depth analysis due to their association with human infections:

• A clade surrounding a fatal human case in Louisiana ([50]; GenBank accession PQ809550) (File S10). Sequences in this subclade are referred to as genotype D1.1.
• A separate, large clade involving transmission between wild birds, poultry, cattle, and a human in Texas (File S10). This subclade contains HA sequences from influenza isolates referred to as genotype B3.13.

StrainHub analysis of the HA subclades of interest show the transmission networks between hosts (Fig. 1). In these figures, the arrowheads represent the direction(s) of the transmission events. The thickness of the lines represents the frequency of the transmission events. Red color is for emphasis. In Fig. 1A, the subclade that contains the virus that led to the fatal human case in Louisiana shows transmission moving bidirectionally from primates (i.e., humans) toward Galliformes and Anseriformes, and back from these 2 groups toward Primates. In Fig. 1B, the human-bovine-avian transmission clade exhibits bidirectional transmission among Galliformes and Primates. In Fig. 1B, there is transmission from Carnivora toward Primates. In Fig. 1B, there is high-frequency transmission from Artiodactyla to Primates.

Curated data from these subclades were carried forward for detailed analyses of natural selection and protein structure.

### Seasonality of H5N1

**Fig. 2.**
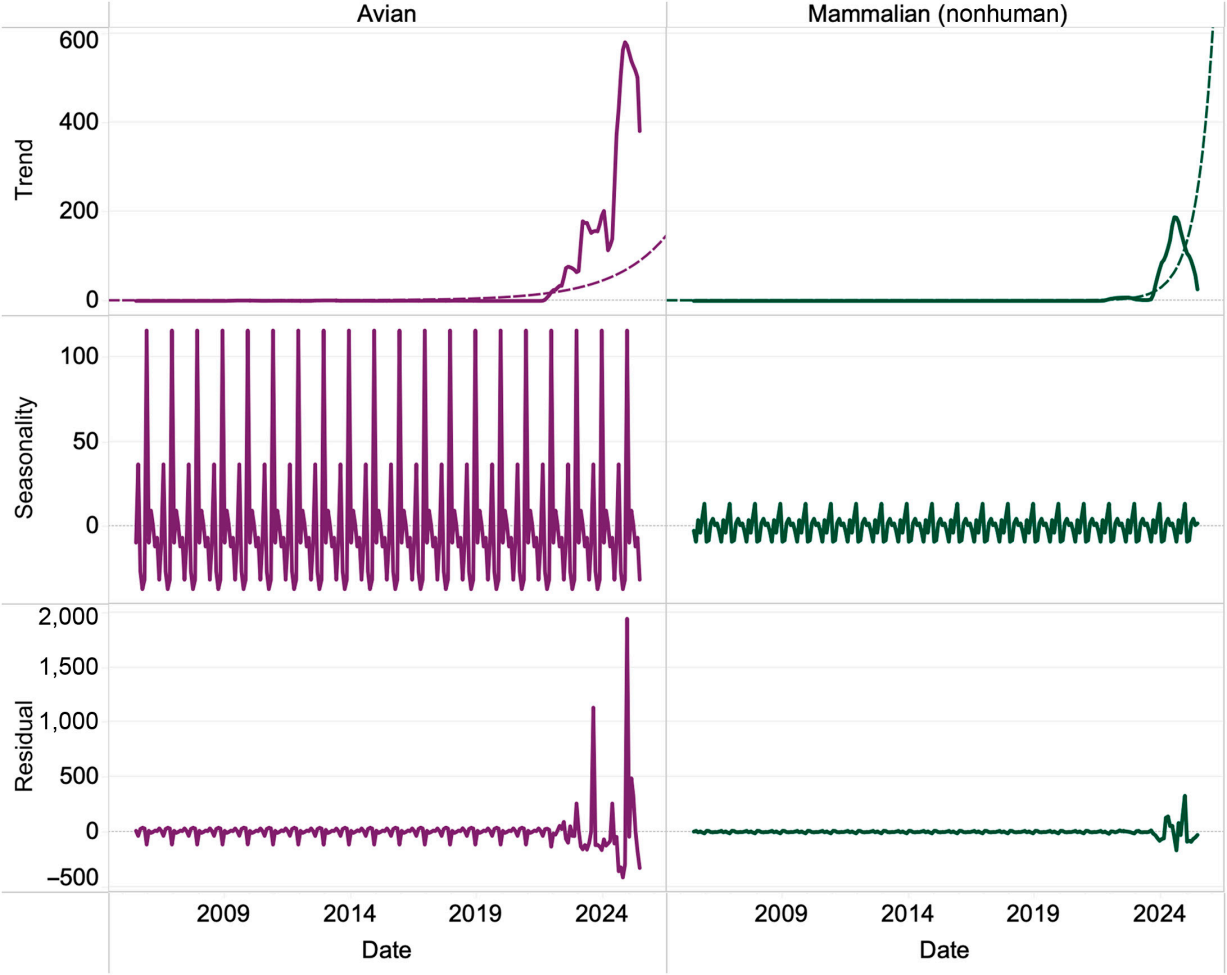
The graph shows seasonal decomposition on United States H5N1 isolates. Data are divided into 2 columns: avian and mammalian (nonhuman) isolates. Rows, from top to bottom, show decompositional trend, the residuals, and final seasonality on the *y*-axis. The *x*-axis shows time spanning 2008 to 2025. Trend and residual spikes can be seen occurring starting in 2021. This analysis shows consistent spikes in cases in winter to spring in the United States.

**Fig. 3.**
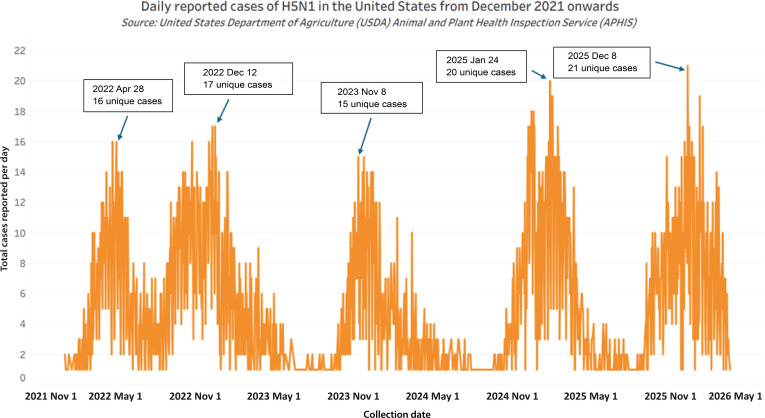
A time-series bar chart depicting the total number of H5N1 nonhuman cases in the United States reported per day over time. These data confirmed detections of H5N1 compiled by the United States Department of Agriculture (USDA) Animal and Plant Health Inspection Service (APHIS) for all animal hosts within the United States from 2021 December 1 to 2026 March 18. These data were sourced from distinct APHIS databases, including one which reports on wild birds (cases starting 2022 January 13), commercial backyard flocks (cases reported starting 2022 February 8), and mammals (cases reported starting 2022 April 24). This analysis reveals a winter-to-spring seasonal pattern of spikes of cases. Peaks in case reporting occurred November to April and notable declines of cases were reported during summer. A maximum was reported on 2025 December 8, during which 25 unique cases were collected and reported to the USDA APHIS databases. Data source: USDA APHIS Avian Influenza Detections database.

To investigate the temporal dynamics of H5N1 outbreaks, we performed seasonal decomposition (Fig. 2) and a time-series analysis (Fig. 3) on reported isolates from avian and mammalian (nonhuman) hosts in the United States. The seasonal decomposition data include 2.3.4.4b clade data and earlier sporadic clades from earlier years. The time-series data focus only on 2.3.4.4b clade data. The seasonal decomposition results indicate that spikes in cases are predominantly driven by outbreaks in avian hosts followed by mammalian hosts (Fig. 2, right). The time-series analysis revealed that peaks in cases occur November to April with an all-time-high peak of outbreak activity in both avian and mammalian (nonhuman) hosts in December of 2025 (Fig. 3, upper right). This analysis indicates that the H5N1 animal pandemic is strengthening into 2025 to 2026.

In terms of sequence data, reporting on GISAID showed that there were 449 recorded isolates in November 2024 and 3,521 recorded isolates in December 2024, indicating a nearly 684% increase in confirmed cases for avian and mammalian (nonhuman) hosts in the United States. This uptick is similarly reported by the USDA APHIS, where there were 552 confirmed cases in November 2024 and 1,189 confirmed cases in December 2024, showing an increase by 115% [51].

### Positive selection

Tables 1 and 2 list the amino acid sites that were found to be under positive selection from HyPhy. No amino acid sites were found to be under positive selection for the protein PB1 within our dataset.

**Table 1.** Rows 1 through 5 contain hemagglutinin (HA) amino acid sites (using sequential site numbering) with a ratio of nonsynonymous to synonymous substitution rates (dN/dS) >1 (model 8; posterior cutoff = 0.95) in the clade surrounding the deceased patient infected with H5N1 in Louisiana. In the rightmost column, we list the mutations observed in these data. Rows 6 through 10 contain HA amino acid sites (using sequential site numbering) with dN/dS >1 (model 8; posterior cutoff = 0.95) in the human-bovine-avian clade. In the rightmost column, we list the mutations observed in these data. Rows 11 through 18 contain polymerase basic 2 (PB2) amino acid sites (using sequential site numbering) with dN/dS >1 (model 8; posterior cutoff = 0.95) in current H5N1 lineages. In the rightmost column, we list the mutations observed in these data.

Protein	Clade	Site	Probability	Mutation(s)
HA	Surrounding the deceased patient infected with H5N1 in Louisiana	140	0.961	N140D/H
HA	Surrounding the deceased patient infected with H5N1 in Louisiana	230	0.969	A230V
HA	Surrounding the deceased patient infected with H5N1 in Louisiana	298	0.970	V298I/L
HA	Surrounding the deceased patient infected with H5N1 in Louisiana	490	0.957	D490N
HA	Surrounding the deceased patient infected with H5N1 in Louisiana	509	0.968	E509G
HA	Human-bovine-avian	11	0.973	V11I
HA	Human-bovine-avian	147	0.999	V147M
HA	Human-bovine-avian	172	0.960	A172T
HA	Human-bovine-avian	250	0.968	K250E/N/R
HA	Human-bovine-avian	526	0.989	I526V
PB2	Current H5N1 lineages	184	0.967	T184A/M/K
PB2	Current H5N1 lineages	249	0.997	E249G/D/K/V
PB2	Current H5N1 lineages	292	0.992	I292L/V/T/M
PB2	Current H5N1 lineages	344	0.999	V344M/A/I
PB2	Current H5N1 lineages	463	0.979	I463M/V
PB2	Current H5N1 lineages	588	0.943 ^a^	A588T/S/V
PB2	Current H5N1 lineages	627	0.999	E627K/V
PB2	Current H5N1 lineages	676	0.967	T676A/I/L/V

^a^ The probability of site 588 is very close to the cutoff and thus was considered in structural analyses.

**Table 2.** Neuraminidase (NA), matrix protein (MP), and polymerase acidic (PA) amino acid sites (using sequential site numbering) with dN/dS >1 (model 8; posterior cutoff = 0.95). In the rightmost column, we list the mutations observed in these data. No amino acid sites were identified to have positive selection in PB1.

Protein	Clade	Site	Probability	Mutation(s)
NA	Subclade 3	16	0.999	V16I/A
NA	Subclade 3	81	0.997	D81A
MP	Current H5N1 lineages	61	1	R61G
MP	Current H5N1 lineages	88	0.999	D88N
PA	Current H5N1 lineages	399	0.947	E399V/G
PA	Current H5N1 lineages	489	0.998	C489S

### Structural analyses reveal increased affinity for mammalian host factors

To understand the functional consequences of mutations revealed by analyses of selection, we performed a series of molecular docking simulations using HADDOCK3 and studied VDW energy as the primary measure of binding affinity prediction.

#### HA docking

**Fig. 4.**
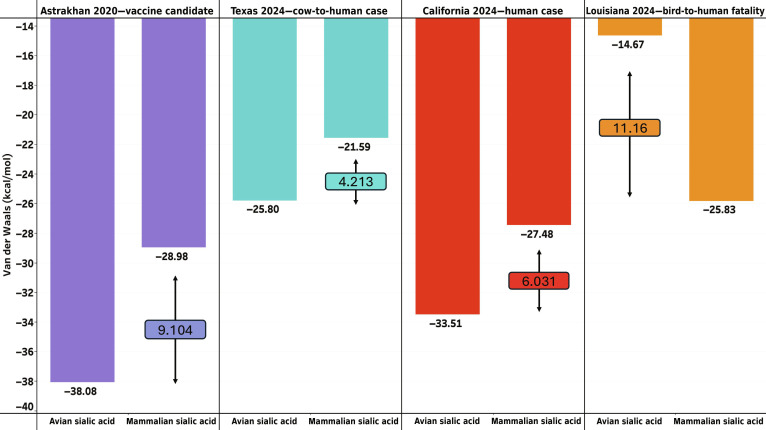
The bar graph shows the results of HADDOCK3 docking simulations for HA binding to avian and human sialic acid (SA) glycans. The *y*-axis represents the Van der Waals’ (VDW) energy, which measures binding affinity, with lower values indicating stronger binding interactions. The *x*-axis categorizes the results based on the type of SA (avian or human). Each color represents an individual sample [blue: EPI1846961 (A/Astrakhan/3212/2020, vaccine candidate), cyan: EPI3171488 (A/Texas/37/2024), red: PQ591824 (A/California/150/2024), and orange: PQ809550 (A/Louisiana/12/2024)]. The numbers boxed between the avian and mammalian bars show the differences between the 2 VDW energies (in kcal/mol). The largest difference is seen in A/Louisiana/12/2024 (orange).

Analysis of the HA–SA docking results indicated a similarity in binding affinity as measured by VDW energy between HA and the mammalian (i.e., human) or avian SA glycan variants in several clades (Fig. 4). In most cases, the avian SA binds HA better than the human SA binds HA. In contrast, in the clade containing the fatal human case in Louisiana (PQ809562, A/Louisiana/12/2024; Fig. 4, orange bars on the right), the human SA binds HA better than the avian SA binds HA.

Across the other viral strains (EPI1846961, A/Astrakhan/3212/2020; PP577947, A/Texas/37/2024; PQ591825, A/California/150/2024; Fig. 4, blue, cyan, and red bars), HA exhibited comparable binding interactions with both human and avian SA.

Additionally, in Fig. S29, we see that the spatial orientation and positioning of SA molecules within the docking simulations remained consistent. This docking experiment predicted that the selected H5N1 clade 2.3.4.4b HA variants retain the capacity for a wide mammalian and avian host range without structural bias. Hence, the virus has evolved host promiscuity.

#### PB2 structures

**Fig. 5.**
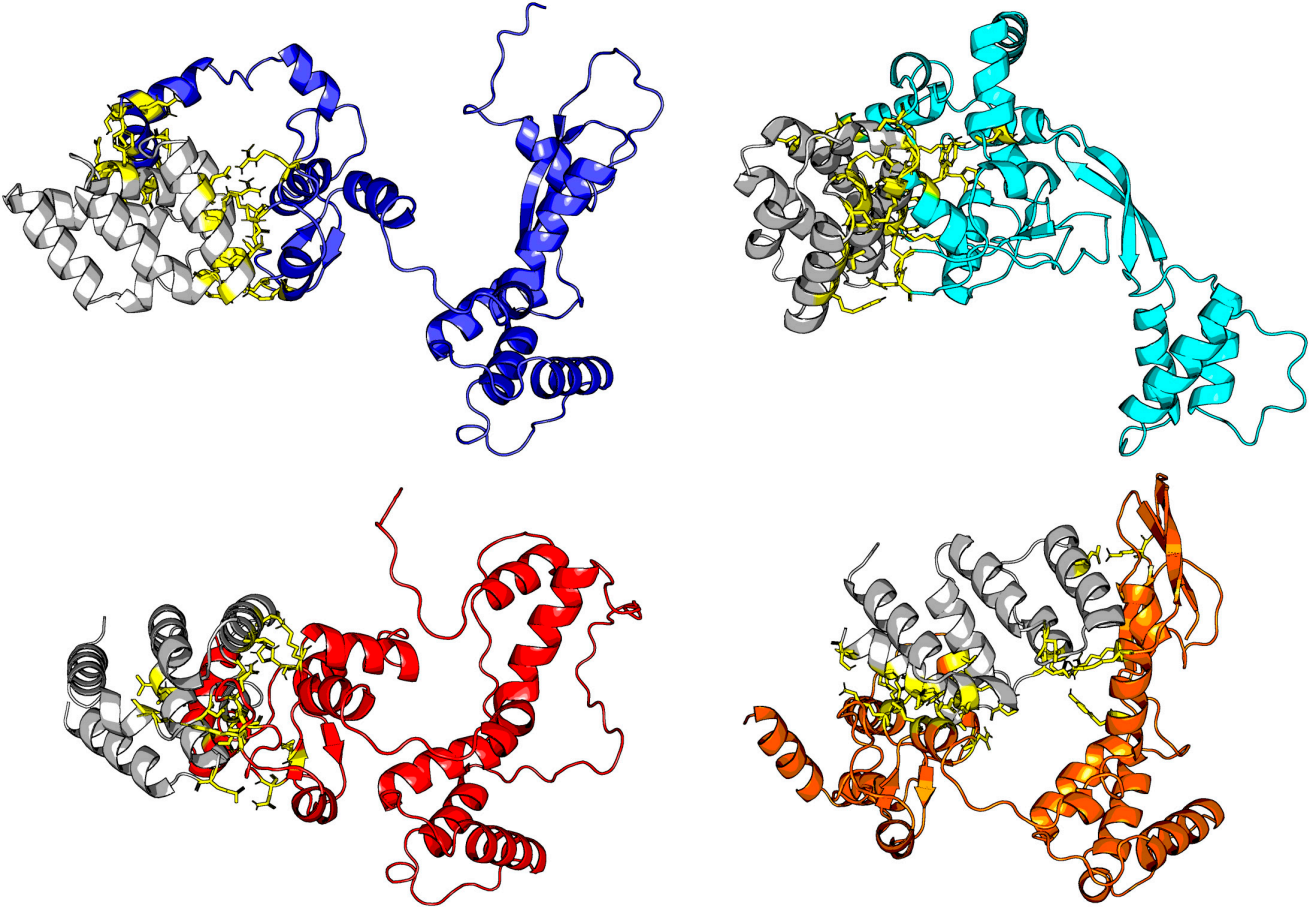
Docking results of MAVS (in gray) against PB2 structures. Each colored PB2 structure represents an individual virus [blue: OQ958041 (A/American Wigeon/South Carolina/22-000345-001/2021, vaccine candidate), cyan: PP577947 (A/Texas/37/2024), red: PQ591825 (A/California/150/2024), and orange: PQ809562 (A/Louisiana/12/2024)].

**Fig. 6.**
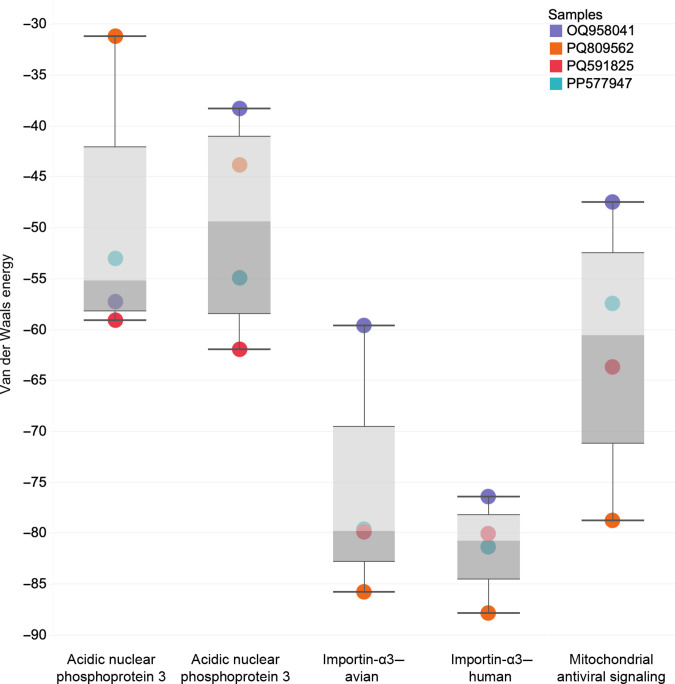
Boxplot graph showing the HADDOCK3 docking results for polymerase basic 2 (PB2) binding to various host immune response proteins. The *y*-axis represents the VDW energy (kcal/mol). We use this metric to assess binding affinity, with lower values indicating stronger interactions. The *x*-axis categorizes the results based on the target proteins: ANP32A (avian and human orthologs), importin-α3 (KPN4; avian and human orthologs), and mitochondrial antiviral signaling (MAVS). Each colored point corresponds to an individual PB2 viral protein [blue: OQ958041 (A/American Wigeon/South Carolina/22-000345-001/2021, vaccine candidate), orange: PQ809562 (A/Louisiana/12/2024), red: PQ591825 (A/California/150/2024), and cyan: PP577947 (A/Texas/37/2024)].

In contrast to the results for HA binding to SA glycans, docking simulations of polymerase PB2 viral protein motifs with host immune response proteins (shown in Fig. 5) showed an increased binding affinity in all recent PB2 strains compared to the vaccine candidate (OQ958041, A/American Wigeon/South Carolina/22-000345-001/2021; Fig. 6). Notably, some recent PB2 strains show stronger binding interactions (lower VDW energy) than the vaccine candidate (OQ958041) for importin-α3 (KPN4) and MAVS. We also see improved binding to human ANP32A as compared to avian ANP32A, which could indicate improved replication within human cells [44]. A/Louisiana/12/2024 presented the greatest difference compared to other strains. These findings predict a potential enhanced ability of recent PB2 variants to inhibit host innate immune signaling pathways, contributing to greater immune evasion [52]. This trend indicates strengthening immune evasion capabilities among recent PB2 variants in both human and avian hosts. This result is also a component of the argument that the virus has evolved host promiscuity.

#### Structural analysis efficacy of commonly used antivirals

**Fig. 7.**
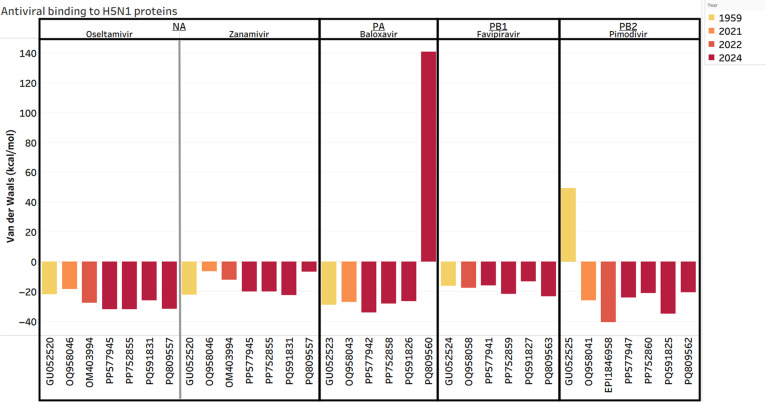
A bar plot showing the binding affinity metric (in VDW energy; *y*-axis) for each protein from each viral isolate to a respective antiviral (*x*-axis). The lower the VDW energy, the stronger the binding affinity. The viral isolates are labeled by their National Center for Biotechnology Information accession ID and refer to the strains A/chicken/Scotland/1959 (GU052520, GU052523, GU052524, and GU052525), A/American Wigeon/South Carolina/22-000345-001/2021 (OQ958046, OQ958043, OQ958058, and OQ958041), A/Astrakhan/3212/2020 (OM403994), A/cattle/Texas/24-009110-004/2024 (PP577945, PP577942, PP577941, and PP577947), A/Texas/37/2024 (PP752855, PP752858, PP752859, and PP752860), A/California/150/2024 (PQ591831, PQ591826, PQ591827, and PQ591825), and A/Louisiana/12/2024 (PQ809557, PQ809560, PQ809563, and PQ809562). Each divided panel shows the protein being represented by the accession IDs (neuraminidase [NA], polymerase acidic [PA], polymerase basic 1 [PB1], and polymerase basic 2 [PB2]) and the antiviral that it is binding to (oseltamivir, zanamivir, baloxavir, favipiravir, and pimodivir). The color gradient from yellow to red signifies the year of collection for each isolate (ranging from 1959 to 2024).

We evaluated the binding strength of approved antiviral drugs to their respective protein targets from recent H5N1 isolates and from an older strain, A/chicken/Scotland/1959, as a benchmark. In Fig. 7, we see continued strength of binding affinity for most antivirals against their respective protein targets even in recent strains. We see a slight decline in binding strength for antivirals zanamivir (NA inhibitor) and a strong decline in binding strength for baloxavir (PA inhibitor) in A/Louisiana/12/2024 (PQ809557 and PQ809560). Notably, A/Louisiana/12/2024 is the isolate that caused the human death in December 2024.

#### Validation of structural analyses

**Fig. 8.**
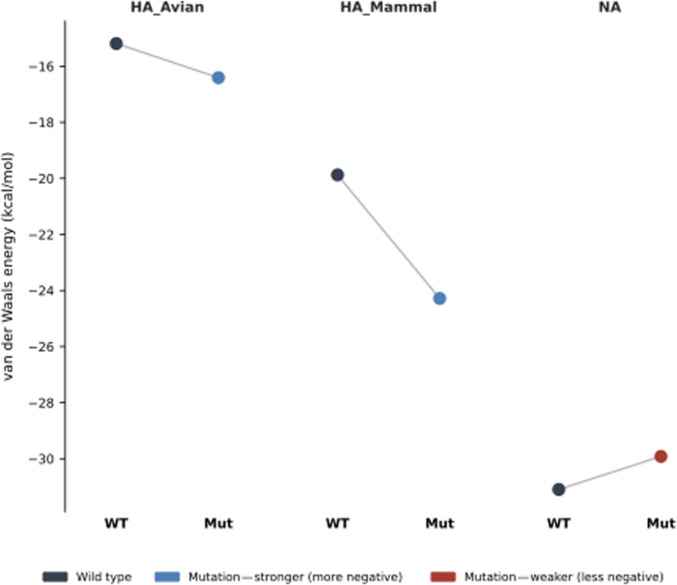
Slopegraph comparing VDW energies (kcal/mol) between wild type (WT) and mutant (Mut) for HA from avian strains (HA_Avian), hemagglutinin from mammalian strains (HA_Mammal), and neuraminidase (NA). Black circles denote WT values; connecting lines indicate the direction and magnitude of change upon mutation. Blue circles indicate mutations that strengthen binding (more negative values). The red circle indicates a mutation that weakens binding (less negative value).

Retrospective benchmarking confirmed that our pipeline successfully anticipates functional shifts in both host range and antiviral susceptibility. In the HA receptor-binding domain, the adapted mutant (9NR5) exhibited improved VDW interaction energies for both avian (16.41 kcal/mol) and mammalian (24.28 kcal/mol) receptors compared to the wild type (9NR2; 15.19 and 19.87 kcal/mol, respectively). This accurately models the “host-range promiscuity” phenotype observed in circulating 2.3.4.4b clades. Furthermore, the pipeline successfully identified the structural disruption caused by the NA H274Y mutation, recording a reduction in oseltamivir binding affinity from 31.09 (wild type: 2HU4) to 29.91 (mutant: 3CL0). These results displayed in Fig. 8 demonstrate that the VDW scoring function is sufficiently sensitive to detect the structural transitions driving H5N1 evolution.

## Discussion

In this study, we combined phylogenetics, natural selection, structural biology, and time-series analyses to track the evolution of H5N1 clade 2.3.4.4b in North America. We assess viral molecular adaptation to avian mammalian hosts. We assess whether there is evolving resistance to small-molecule antivirals.

We performed decomposition of seasonality and empirical time-series analyses to assess whether there are surges in cases of H5N1 infections across avian and mammalian hosts. Our work is a use case of rapid computational structural predictions for identifying functional changes in mutated viral sequences that adds a new functional component to molecular epidemiology. Our analyses indicate the evolution of viral promiscuity to infect both avian and mammalian hosts and blocking of the innate immune response of mammalian cells. Moreover, we discover that there have been, since 2021, seasonal surges in H5N1 infection in both avian and mammalian hosts in the United States.

### Trends in seasonality

Our decomposition of seasonality and empirical time-series analyses reveal a profound shift in the epidemiology of H5N1, characterized by an increase in cases since 2021 with more cases in the winter to spring in the United States. The dramatic surge in H5N1 cases corresponds directly with the introduction of H5N1 clade 2.3.4.4b to the United States, adaptation, and spread in at least 2 distinct subclades among many avian and mammalian wild and domestic hosts [53].

### Phylogenetic discernment of lineages of zoonotic interest and analyses of natural selection

The rapid expansion of H5N1 clade 2.3.4.4b has generated a massive volume of genomic data. To navigate this large dataset, we employed a phylogenetic approach to narrow our focus from broad surveillance to clades in which zoonosis was occurring. We then conducted experiments to understand the specific molecular mechanisms driving each zoonosis event. By reconstructing the evolutionary history of recent North American isolates, we identified 2 distinct subclades of high zoonotic interest: the genotype D1.1 lineage associated with the fatal human case in Louisiana and the genotype B3.13 lineage driving the bovine-human transmission events [51]. We used StrainHub network analysis (Fig. 1) to further validate this focus. The StrainHub analysis reveals distinct transmission dynamics in each subclade. The Louisiana subclade is characterized largely by avian-primate exchanges, whereas the other subclade exhibited high-frequency transmission between artiodactyls and primates.

Once these lineages were isolated, we applied natural selection analysis using HyPhy (dN/dS) to identify specific amino acid sites undergoing positive selection and potentially driving adaptation. In the HA protein, we identified distinct selective pressures between the 2 subclades. The Louisiana-associated lineage showed positive selection at sites such as A230V, a mutation historically linked to stability and immune escape [54]. Conversely, the bovine lineage exhibited selection at site A172T, a mutation often associated with increased replication in mammalian cell culture [55].

In the viral polymerase complex, our analysis highlighted a positive selection in the PB2 subunit in several sites. Highlights include E249G and E627K. For E249G, a historical study of H5N1 viruses demonstrates that this mutation improves replication in human cells [56]. Moreover, E627K is a well-studied mutation underlying the transmission of avian-hosted lineages H5N1 to mammals [57]. E627K also increases the replication and virulence of the H5N1 in mammals [58]. One human isolate in our data that bears E627K [GenBank: PP577947 A/Texas/37/2024(H5N1)] has been shown in experiments to transmit via respiratory droplets among ferrets, leading to mortality in most of the ferrets [59].

In NA, positive selection was detected at sites 16 and 81. While these sites are evolving, they are not located within or directly adjacent to the highly conserved active site of the NA enzyme, where inhibitors like oseltamivir and zanamivir bind. This suggests that the evolutionary changes at these positions are not likely to confer direct resistance to these drugs.

In the MP protein, positive selection was found at sites 61 and 88. However, resistance to adamantane-class MP inhibitors is primarily associated with mutations within the transmembrane domain and ion channel pore, most notably at site 31 (S31N) [60]. The sites under selection fall outside this critical region, indicating that these mutations are unlikely to impact MP inhibitor effectiveness.

For the polymerase complex, positive selection was observed at sites 399 and 489 in the PA subunit. The primary resistance mechanism to the cap-dependent endonuclease inhibitor, baloxavir marboxil, involves a key mutation at site 38 (I38T) [28,61]. As the sites we identified are distinct from the known resistance-conferring mutations, their selection does not directly imply a loss of baloxavir efficacy.

Notably, we found a lack of positive selection in the PB1 subunit in our dataset of recent North American H5N1 isolates. This result suggests that the PB1 protein, a core component of the RNA-dependent RNA polymerase, is currently under strong evolutionary constraint within this viral population to maintain its essential function. This high degree of conservation is particularly notable when contrasted with previous studies that have identified specific PB1 mutations associated with increased virulence and mammalian adaptation in other H5N1 lineages. For instance, substitutions like N105S have been shown to enhance polymerase activity in mammalian cells, contributing to increased pathogenicity [62]. Furthermore, while resistance to the PB1-targeting antiviral favipiravir is rare in natural isolates, specific mutations such as K229R have been generated in vitro and can confer reduced susceptibility [63]. The fact that our analysis did not detect positive selection at these or any other sites suggests that, within the evolutionary pressures currently acting on H5N1 in North America, adaptations in PB1 are not a primary driver of host shifts or emerging antiviral resistance.

Residues that have shown mutations under selection were used to define areas of interest of docking simulations. This resulted in the selection of the HA receptor-binding domain and several domains within PB2, such as the amino terminal and the 627 domain [64], along with antiviral binding pockets for NA, PA, PB1, and PB2. While mutations of interest were not directly involved as active binding residues in the docking simulations, these mutations were present near the active binding residues. These mutations cause conformation changes, changes in charges and side chain sizes, and/or effect residue polarity [65]. Mutations of interest are elaborated below.

### Host–protein interactions

HA binding to SA remains a central determinant of host specificity. Previous studies have established that avian-adapted influenza strains preferentially bind α2,3-linked SA, whereas human-adapted strains favor α2,6-linked SA [24]. Comparison of mutations within the HA RBD to active binding residues reveals that while the mutations are not directly involved in receptor binding, several sites that are under selection (e.g. 140, 147, and 230) lie within 10 amino acids of key binding sites. The proximity can introduce steric effects that influence binding affinity, alter host-receptor specificity, and increase host promiscuity. Dadonaite et al. [66] point out sets of mutations of interest they discovered in H5N1 HA in combined computational and pseudoparticle experiments for receptor binding, HA stability, and immune escape mutations. We compared our mutations of interest from Tables 1 and 2 with the mutations of interest in Dadonaite et al. [66], with adjustments for numbering conventions. We did not find exact overlaps in sites, but we did find overlaps in domains, while we saw no dramatic differences with the RBD of our selected isolates for the structural analysis. However, we observed a drastic difference in binding affinity of A/Louisiana/12/2024 HA protein binding to mammalian SA as compared to avian SA. This increase in binding affinity to mammalian SA could signify a greater preference to mammalian host cells.

This promiscuity afforded by the adaptation of binding to a variety of receptors indicates that these strains retain the potential for widening zoonotic transmission and subsequent increases in mutation and reassortment events. Empirical studies have shown increased viral replication of the H5N1 virus isolated from cattle in 2024 in a variety of mammalian cells including human and canid [67]. The structural consistency in HA–SA interactions across different strains indicates that receptor binding alone may not be the primary barrier to human adaptation in these viruses, and alternative host factors have been proposed to facilitate influenza infection. Recent literature has highlighted the role of the multibasic cleavage site in expanding viral tropism, and studies in other viruses, such as SARS-CoV-2, have demonstrated that neuropilin-1 can act as an auxiliary entry factor [68,69]. While some evidence suggests that certain HA-derived peptides may interact with host proteoglycans in a sialic-acid-independent manner, the significance of these pathways in the context of whole-virus H5N1 host-range expansion remains uncertain [70]. As noted by Azeem et al. [71], sialic acid remains the predominant pathway for H5N1 entry. Additional factors such as polymerase function and host immune evasion are likely to contribute to host adaptation [72].

PB2, a key component of the viral RNA polymerase complex, has been shown to influence host adaptation by interacting with cellular factors involved in nuclear trafficking and immune signaling [73,74]. Our docking analysis highlights strong PB2 interactions with MAVS and KPN4, particularly in recent strains, which bind much stronger to these proteins than the vaccine candidate (OQ958041) does. MAVS protein is a critical component of the hosts’ antiviral response, and an increased PB2 binding affinity enables immune evasion by hindering interferon signaling [43,75]. Moreover, Octaviani et al. [67] show a lack of retinoic acid-inducible gene I (RIG-1) levels in infected cells by 2024 viral strains. This result indicates antiviral signal antagonism by recent strains of the virus. RIG-1 is part of the innate immune response that causes cells to turn into an antiviral state and MAVS initiates this signaling pathway for RIG-1 [76]. The results from Octaviani et al. [67] strengthen our findings as increased binding of MAVS impedes its signaling. Similarly, KPN4 and ANP32A mediate nuclear import of viral ribonucleoproteins. Improved binding between PB2 and KPN4 shows improved capability of nucleus localization in host cells to initiate influenza viral replication [25]. Enhanced PB2–ANP32A interactions improve viral replication efficiency in mammalian cells as it has been shown to promote viral replication in Yu et al. [77]. Our results show a slight weakening of binding affinity of PB2 with ANP32A, the viral replication enhancer, in recent strains. However, we see an increased binding affinity of PB2 with the nuclear transporter KNP4 and the immune response protein MAVS (i.e., in the A/Louisiana/12/2024 strain that caused a fatality as compared to the vaccine candidate strain).

### Antiviral interactions

A crucial aspect of pandemic preparedness is understanding the continued effectiveness of existing medical countermeasures. Our positive selection analysis did not find any amino acid sites in respective proteins that lead to changes within their binding pockets for these antivirals. On the other hand, our structural analysis of antiviral binding provided insights into this question. We found that the binding affinity for most antivirals, including the widely used NA inhibitor oseltamivir, remained largely stable across recent H5N1 isolates compared to an older reference strain. However, we observed a notable decrease in the predicted binding affinity for zanamivir (NA inhibitor) and baloxavir (PA inhibitor) for the A/Louisiana/12/2024 isolate, which was associated with a fatal human infection. No public information is available regarding the type of treatment used on the patient. The Centers for Disease Control and Prevention states primary treatment of influenza infections with oseltamivir (NA inhibitor) and then addition of baloxavir for persistent infection [49]. This finding highlights the potential for resistance to emerge on a strain-specific basis and could lead to decrease in treatment options. This discovery highlights the importance of continuous surveillance to monitor for increased spread of these resistant mutants [78]. While our data support the continued primary use of oseltamivir in North America, the reduced predicted efficacy of other antivirals (i.e., zanamivir and baloxavir) against a highly pathogenic human isolate is an important concern that warrants further investigation.

### Limitations

This study has limitations inherent to its computational nature. Our findings on binding affinity and selection are predictive and will benefit and inspire empirical research through in vitro and in vivo experiments. Nevertheless, we validated the work in an experiment herein and within several papers: Ford et al. [79], Ford et al. 2023 [80], Yasa et al. 2024 [81], and Ford et al. 2025 [10].

The workflow we have established in this paper expands on this body of work by including large-scale phylogenetics, analyses of natural selection, and artificial-intelligence-driven structural biology, joining a powerful set of tools that bring molecular disease surveillance forward to functional molecular disease intelligence. As sequence data become available and artificial intelligence more powerful, this approach can be rapidly deployed to assess new pathogens and countermeasures, providing early warnings and guiding public health responses before a crisis escalates.

## Conclusion

This study not only characterizes the expanding threat of H5N1 clade 2.3.4.4b but, more critically, demonstrates the potential of an integrated computational framework for outbreak preparedness and response. Our experiment shows that combining phylogenetic surveillance with high-throughput structural docking can predict complex shifts in viral functionality, specifically host promiscuity, immune evasion, and antiviral resistance, with recent empirical results validating our results.

While empirical techniques such as binding assays and animal models remain the gold standard for confirmation, they are inherently resource intensive and time consuming, often lagging behind the rapid rate of viral evolution. In contrast, the workflow presented here allows for the near real-time translation of genomic data into phenotypic predictions. By simulating protein–host interactions in silico, we can identify high-risk variants and functional anomalies before they are fully characterized in the laboratory. This capacity to predict changes in host adaptability and immune response demonstrates that computational structural biology workflows can serve as a vital early warning system, shifting the paradigm from reactive surveillance to proactive risk assessment.

Future research integrating structural modeling with functional assays will be crucial for validating these computational predictions and improving our understanding of influenza A H5N1 host adaptation mechanisms and therapeutics [10].

## Data Availability

Raw sequence data, alignments, and results from phylogenetics and analyses of selection, predicted protein structures, and protein–protein binding data are available on GitHub at https://github.com/colbyford/Influenza_A_2.3.4.4b_Analyses. Code for running the analyses used in this study is available on GitHub at https://github.com/colbyford/Influenza_A_2.3.4.4b_Analyses.
