# Supplementary material for: Computational Structural Analysis Predicts Host-Range Promiscuity and Antiviral Resistance in North American H5N1 Lineages
Source: Comput Struct Biotechnol J. 2026 May 8;35(1):0066. doi: 10.34133/csbj.0066 (PMC13153456; doi:10.34133/csbj.0066)
Supplement: Supplementary 1 — Files S1 to S23 Figs. S24 to S30 [file csbj.0066.f1.zip › README.docx]

Below are supplemental files for

Analyses of phylogenetics, natural selection, and protein structure of clade 2.3.4.4b H5N1 Influenza A reveal that recent viral lineages have evolved promiscuity in host range and improved replication in mammals in North America

Daniel Janies^1,2,*^, Kary Ocaña^3^, Sayal Guirales-Medrano^1,2^, Khaled Obied^1,2^,

Rachel Alexander^1,2^, and Colby T. Ford^1,2,4,5^

**Affiliations:**

1. Center for Computational Intelligence to Predict Health and Environmental Risks (CIPHER), University of North Carolina at Charlotte, Charlotte, NC, USA
2. Department of Bioinformatics and Genomics, University of North Carolina at Charlotte, Charlotte, NC, USA
3. National Laboratory of Scientific Computing, Rio de Janeiro, Brazil
4. School of Data Science, University of North Carolina at Charlotte, Charlotte, NC, USA
5. Tuple LLC, Charlotte, NC, USA

*Please direct questions to the corresponding author: [djanies@charlotte.edu](mailto:djanies@charlotte.edu)

—------------------------------------------------------------------------------------------------------------------

supplemental_data_file_1.csv

Legend

Merger of data from the United States Centers for Disease Control and Prevention (CDC) and The World Organization for Animal Health (WOAH) for H5N1 infections in mammals and birds in the USA 1/12/22 to 3/1/25.

—------------------------------------------------------------------------------------------------------------------

supplemental_data_file_2.csv

Legend

Merger of data from the Canadian Food Inspection Agency; the Government of Canada;

Environment and Climate Change Canada; Canadian Wildlife Health Cooperative; Public Health Agency of Canada; Parks Canada; Canadian Animal Health Surveillance System; Alberta Agriculture, Forestry, and Rural Economic Development Laboratory, Animal Health Laboratory, Laboratory Services Division; University of Guelph, Atlantic Veterinary College – Veterinary Laboratory and Pathology Services; Manitoba Agriculture - Veterinary Diagnostic Services; Ministère de l’Agriculture, des Pêcheries et de l’Alimentation du Québec - Laboratoire de santé animale; New Brunswick Department of Agriculture, Aquaculture and Fisheries - Veterinary Laboratory and Pathology Services; Prairie Diagnostic Services; Government of Alberta; Government of British Columbia; Government of Manitoba; Government of New Brunswick; Government of Newfoundland and Labrador; Government of Northwest Territories; Government of Nova Scotia; Government of Nunavut; Government of Ontario; Government of Quebec; Government of Saskatchewan; Government of Yukon, and The World Organization for Animal Health (WOAH) for H5N1 infections in mammals and birds in the USA 12/9/2021 to 2/13/2025.

—------------------------------------------------------------------------------------------------------------------

supplemental_data_file_3.csv

Legend

Accession ID, year of isolation, host of isolation, and strain name for 9,359 sequence from the aforementioned dataset of HA sequence data that contained clade 2.3.4.4b and background data.

—------------------------------------------------------------------------------------------------------------------

supplemental_data_file_4.nexus

/Users/djanies/UNC Charlotte Dropbox/Daniel Janies/H5N1-2024/segments/HA/Pulit-Penaloza/new-new-HA-Dec-30/full-dataset-9359/tnt/v3/H5N1_N2_HA_nuc_djv28_Pulit-Penaloza-blast-LA-NM_noDup.trim copy 2.nexus.

Legend

Curated multiple sequence alignment of 9359 Hemagglutinin nucleotide sequences for H5N1 aligned to 1704 columns.

—------------------------------------------------------------------------------------------------------------------

supplemental_data_file_5.nexus

Legend

Curated multiple sequence alignment of 3415 Polymerase Basic 2 nucleotide sequences for H5N1 aligned to 2281 columns.

—------------------------------------------------------------------------------------------------------------------

supplemental_data_file_6.phy

Legend

Curated multiple sequence alignment of 331 Hemagglutinin nucleotide sequences for H5N1 aligned to 1701 columns (the avian-human subclade associated with the fatal human case in Louisiana).

—------------------------------------------------------------------------------------------------------------------

supplemental_data_file_7.phy

Legend

Curated multiple sequence alignment of 767 Hemagglutinin nucleotide sequences for H5N1 aligned to 1701 columns (the bovine-avian-human subclade).

—------------------------------------------------------------------------------------------------------------------

supplemental_data_file_8.phy

Legend

Curated multiple sequence alignment of the subclade of interest for zoonotic events containing 1528 Polymerase Basic 2 nucleotide sequences for H5N1 aligned to 2277 columns.

——---------------------------------------------------------------------------------------------------------------

supplemental_data_file_9.log

Legend

TNT log for Hemagglutinin H5N1 nucleotide alignment of 9359 sequences and 1704 positions.

——---------------------------------------------------------------------------------------------------------------

supplemental_data_file_10.log

Legend

TNT log file for tree 0 Hemagluttinin H5N1 nucleotide alignment of 9359 sequences and 1704 positions in which exemplar sequences from zoonotic clades of interest are marked with <---- to illustrate how distant they are in the tree.

——---------------------------------------------------------------------------------------------------------------

supplemental_data_file_11.log

Legend

TNT log file or the search of the Polymerase basic 2 nucleotide data for H5N1 containing 3415 sequences and 2281 aligned positions containing heuristically parsimonious trees of 12133 steps.

——---------------------------------------------------------------------------------------------------------------

supplemental_data_file_12.log

Legend

TNT log containing the first tree with key sequences marked with “<---” which we used to select a subclade of interest based on the position of isolates of interest.

—------------------------------------------------------------------------------------------------------------------

supplemental_data_file_13.txt

Legend

HyPhy output for analyses of selection on supplemental_data_file_6.phy (the avian-human subclade associated with the fatal human case in Louisiana)

—------------------------------------------------------------------------------------------------------------------

supplemental_data_file_14.txt

Legend

HyPhy output for analyses of selection on supplemental_data_file_7.phy

(the bovine-avian-human subclade)

—------------------------------------------------------------------------------------------------------------------

supplemental_data_file_15.txt

Legend

HyPhy output for analyses of selection on supplemental_data_file_8.phy (subclade of interest for zoonotic events in PB2 data).

—------------------------------------------------------------------------------------------------------------------

supplemental_data_table_16.pdf

Legend

Acknowledgement that some viral HA sequences and associated metadata in this dataset are published in GISAID’s EpiFlu database. Other viral HA sequences and associated metadata in this dataset are in NIH's Genbank.

—------------------------------------------------------------------------------------------------------------------

supplemental_data_table_17.pdf

Acknowledgement that some viral PB2 sequences and associated metadata in this dataset are published in GISAID’s EpiFlu database. Other viral PB2 sequences and associated metadata in this dataset are in NIH's Genbank.

Supplemental_data_file_18.nexus

Legend

Curated multiple sequence alignment of 22858 Matrix Protein 2 nucleotide sequences for H5N1 aligned to 294 columns.

Supplemental_data_file_19.nexus

Legend

Curated multiple sequence alignment of 257 Neuraminidase nucleotide sequences for H5N1 aligned to 1410 columns.

Supplemental_data_file_20.nexus

Legend

Curated multiple sequence alignment of 429 Polymerase Acid nucleotide sequences for H5N1 aligned to 2148 columns.

Supplemental_data_file_21.nexus

Legend

Curated multiple sequence alignment of 9841 Polymerase Basic 1 nucleotide sequences for H5N1 aligned to 2274 columns.

supplemental_data_file_22.phy

Legend

Curated multiple sequence alignment of the subclade of interest for zoonotic events containing 22858 Matrix protein 2 nucleotide sequences for H5N1 aligned to 294 columns.

Supplemental_data_file_23.phy

Legend

Curated multiple sequence alignment of the subclade of interest for zoonotic events containing 9841 Polymerase Basic 1 nucleotide sequences for H5N1 aligned to 2274 columns.

Supplemental_fig_24.png

Legend

Docking results of ANP32A (in magenta) against PB2 structures. Each colored PB2 structure represents an individual virus [Blue: OQ958041 (A/American Wigeon/South Carolina/22-000345-001/2021, vaccine candidate), Cyan: PP577947 (A/Texas/37/2024), Red: PQ591825 (A/California/150/2024), and Orange: PQ809562 (A/Louisiana/12/2024)].

Supplemental_fig_25.png

Legend

Docking results of avian KPN4 (in green) against PB2 structures. Each colored PB2 structure represents an individual virus [Blue: OQ958041 (A/American Wigeon/South Carolina/22-000345-001/2021, vaccine candidate), Cyan: PP577947 (A/Texas/37/2024), Red: PQ591825 (A/California/150/2024), and Orange: PQ809562 (A/Louisiana/12/2024)].

Supplemental_fig_26.png

Legend

Docking of the neuraminidase (NA) protein for A/chicken/Scotland/1959 (Yellow), A/American Wigeon/South Carolina/22-000345-001/2021 (Purple), A/Astrakhan/3212/2020 (Blue), A/cattle/Texas/24-009110-004/2024 (Green), A/Texas/37/2024 (Cyan), A/California/150/2024 (Red), A/Louisiana/12/2024 (Orange) against the antiviral drug Oseltamivir.

Supplemental_fig_27.png

Legend

Docking of the Polymerase Acid (PA) protein for A/chicken/Scotland/1959 (Yellow), A/American Wigeon/South Carolina/22-000345-001/2021 (Purple), A/Astrakhan/3212/2020 (Blue), A/cattle/Texas/24-009110-004/2024 (Green), A/Texas/37/2024 (Cyan), A/California/150/2024 (Red), A/Louisiana/12/2024 (Orange) against the antiviral drug Baloxavir.

Supplemental_fig_28.png

Legend

A boxplot graph showing the results of HADDOCK3 docking simulations for Hemagglutinin (HA) binding to avian and human sialic acid (SA) glycans. The y-axis represents the Van der Waals’ energy, which measures binding affinity, with lower values indicating stronger binding interactions. The x-axis categorizes the results based on the type of SA (avian or human). Each colored point represents an individual sample [Blue: EPI1846961 (A/Astrakhan/3212/2020, vaccine candidate), Cyan: EPI3171488 (A/Texas/37/2024), Red: PQ591824 (A/California/150/2024), and Orange: PQ809550 (A/Louisiana/12/2024)], and their corresponding energies are plotted for both avian and human SA. The figure suggests that HA binding energies do not show a significant preference between human and avian SA, as the distributions overlap considerably.

Supplemental_fig_29.png

Legend

Docking results of avian (green) and human (magenta) sialic acid (SA) with each Hemagglutinin (HA) sample. Each colored HA structure represents an individual virus [Blue: EPI1846961 (​​A/Astrakhan/3212/2020, vaccine candidate), Cyan: EPI3171488 (A/Texas/37/2024), Red: PQ591824 (A/California/150/2024), and Orange: PQ809550 (A/Louisiana/12/2024)]. The analysis reveals minimal variation in the interaction patterns between human and avian SA, as indicated by the similar binding orientations and positions of the SA molecules. Additionally, the binding interactions remain consistent across different HA samples for each type of SA, suggesting a lack of significant structural preference for either human or avian SA.

Supplemental_fig_30.png

Legend

This bar graph contains a representation of the total number of states with confirmed cases of H5N1 in livestock, poultry, mammals, and wild birds in the United States from 01/01/2024 to 06/05/2025. The data for this was sourced from the United States Department of Agriculture’s APHIS program. The y-axis measures the total number of states with confirmed cases of H5N1, and the x-axis shows the month each case was reported and detected. Each bar is labeled for clarity.
