# Supplementary material for: Computational Structural Analysis Predicts Host-Range Promiscuity and Antiviral Resistance in North American H5N1 Lineages
Source: Comput Struct Biotechnol J. 2026 May 8;35(1):0066. doi: 10.34133/csbj.0066 (PMC13153456; doi:10.34133/csbj.0066)
Supplement: Supplementary 1 — Files S1 to S23 Figs. S24 to S30 [file csbj.0066.f1.zip › supplemental_data_file_16.pdf]

SUPPLEMENTAL TABLE

Data Availability

GISAID Identifier: EPI_SET_250228cs
doi: 10.55876/gis8.250228cs

Some genome sequences and associated metadata in this dataset are published in GISAID's EpiFlu database. To view the contributors of each individual sequence with details such as accession number, Virus name, Collection date, Originating Lab and Submitting Lab and the list of Authors, visit 10.55876/gis8.250228cs

Data Snapshot

- EPI_SET_250228cs is composed of 9063 individual genome segments;
- The collection dates range from 1997-01-01 to 2024-11-17;
- Data were collected in 90 countries and territories.
