# Supplementary figures and images for: Computational Structural Analysis Predicts Host-Range Promiscuity and Antiviral Resistance in North American H5N1 Lineages
Source: Comput Struct Biotechnol J. 2026 May 8;35(1):0066. doi: 10.34133/csbj.0066 (PMC13153456; doi:10.34133/csbj.0066)

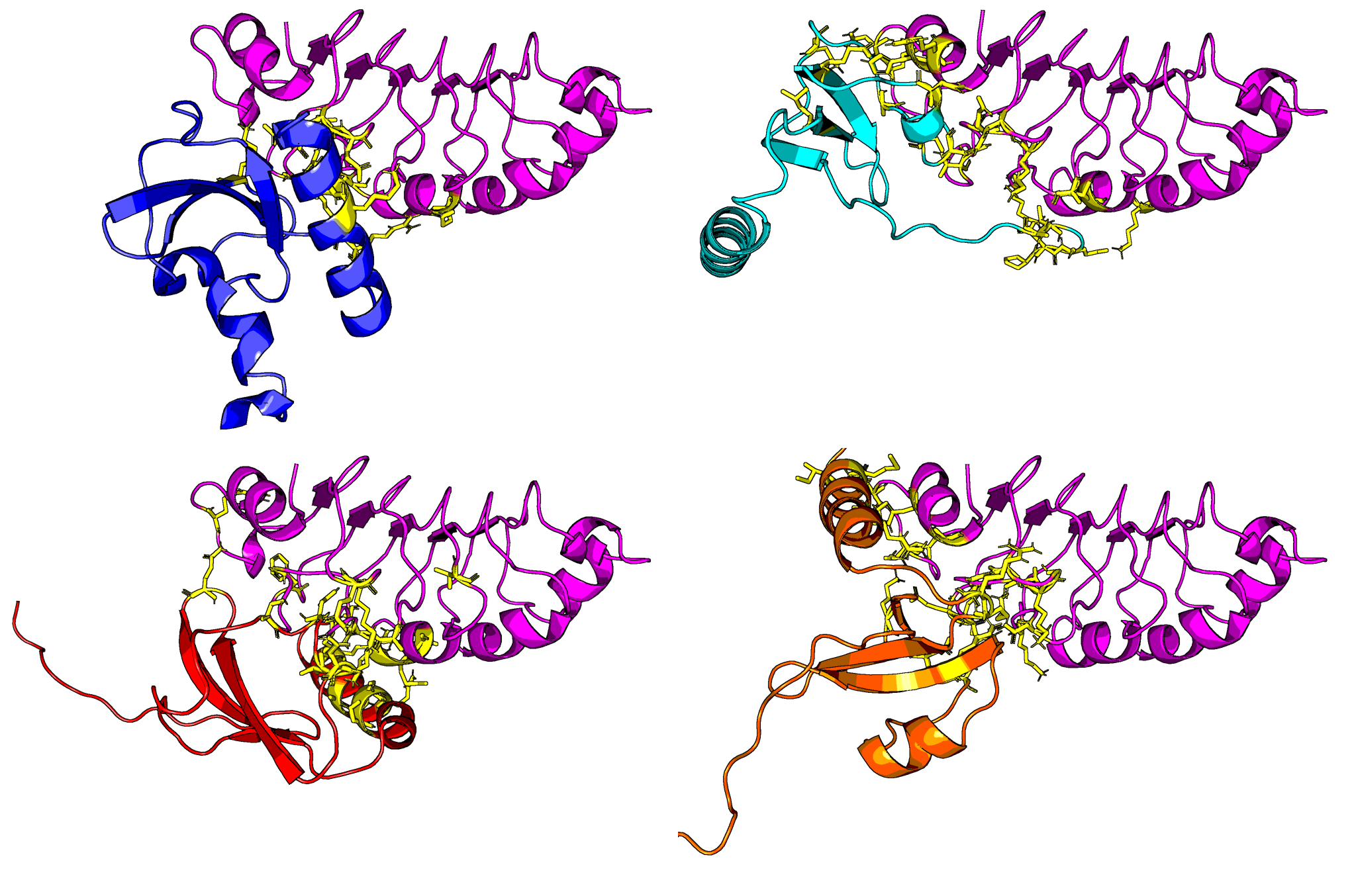

Supplement: Supplementary 1 — Files S1 to S23 Figs. S24 to S30 [file csbj.0066.f1.zip › supplemental_fig_24.png]

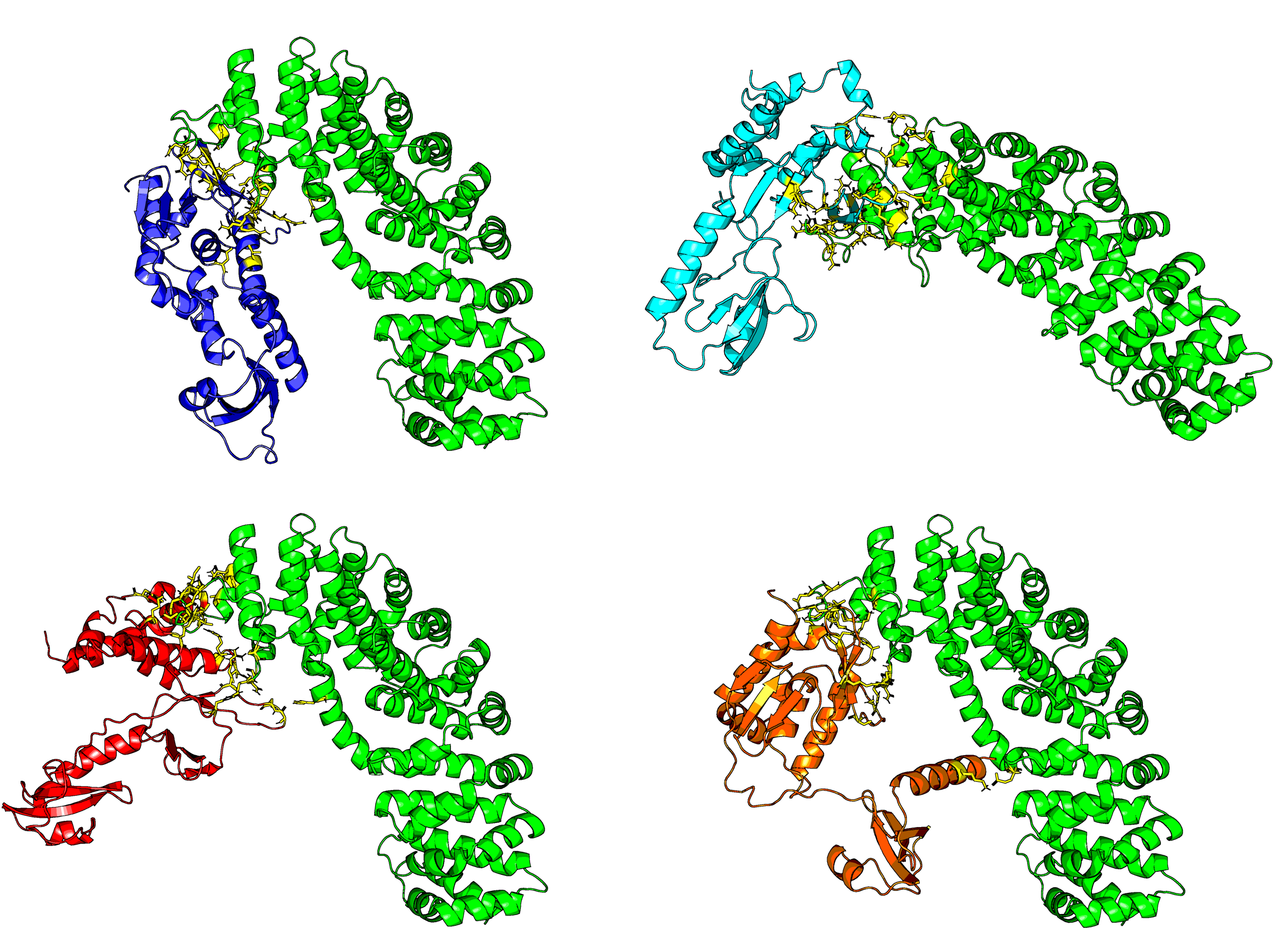

Supplement: Supplementary 1 — Files S1 to S23 Figs. S24 to S30 [file csbj.0066.f1.zip › supplemental_fig_25.png]

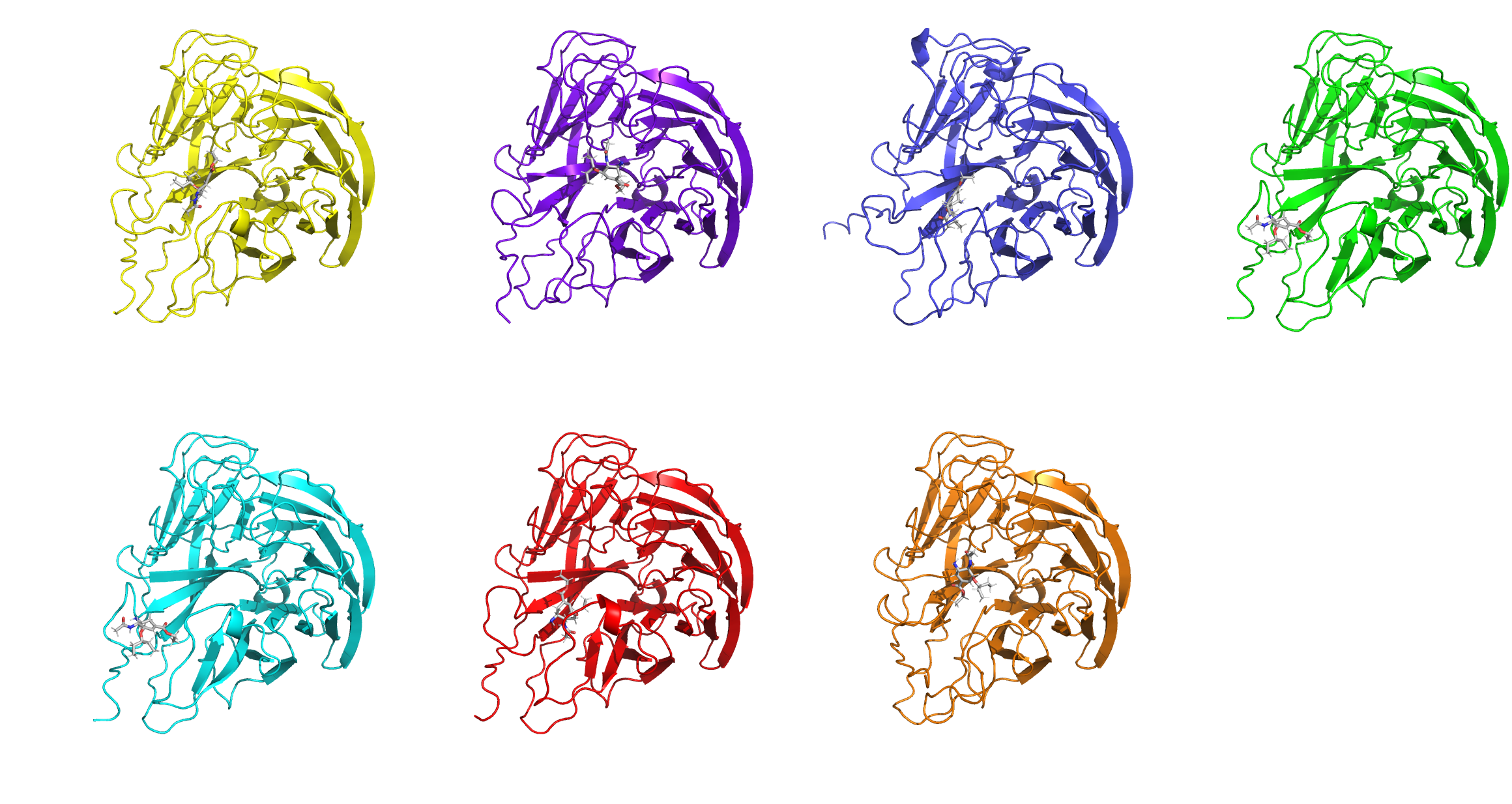

Supplement: Supplementary 1 — Files S1 to S23 Figs. S24 to S30 [file csbj.0066.f1.zip › supplemental_fig_26.png]

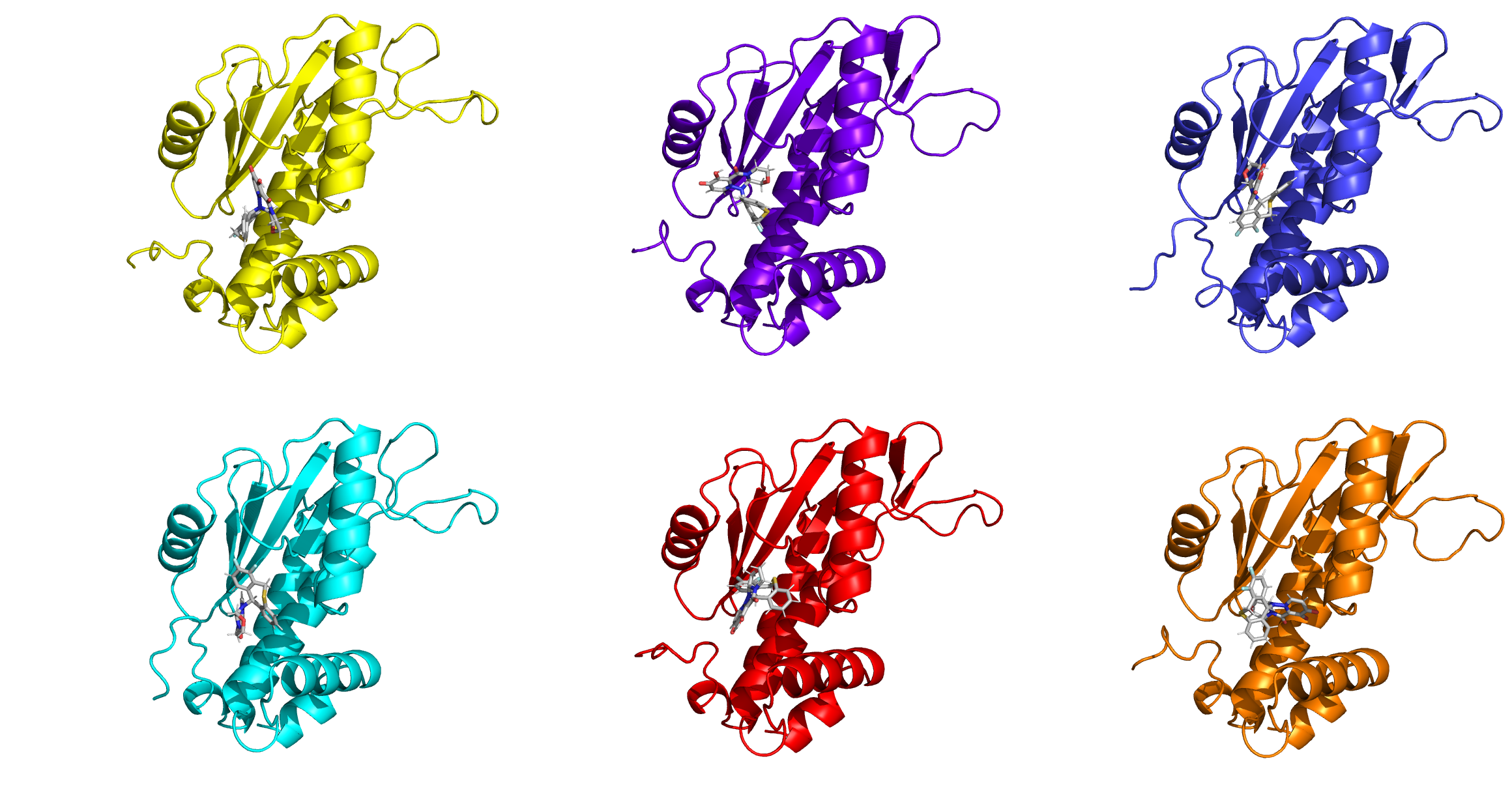

Supplement: Supplementary 1 — Files S1 to S23 Figs. S24 to S30 [file csbj.0066.f1.zip › supplemental_fig_27.png]

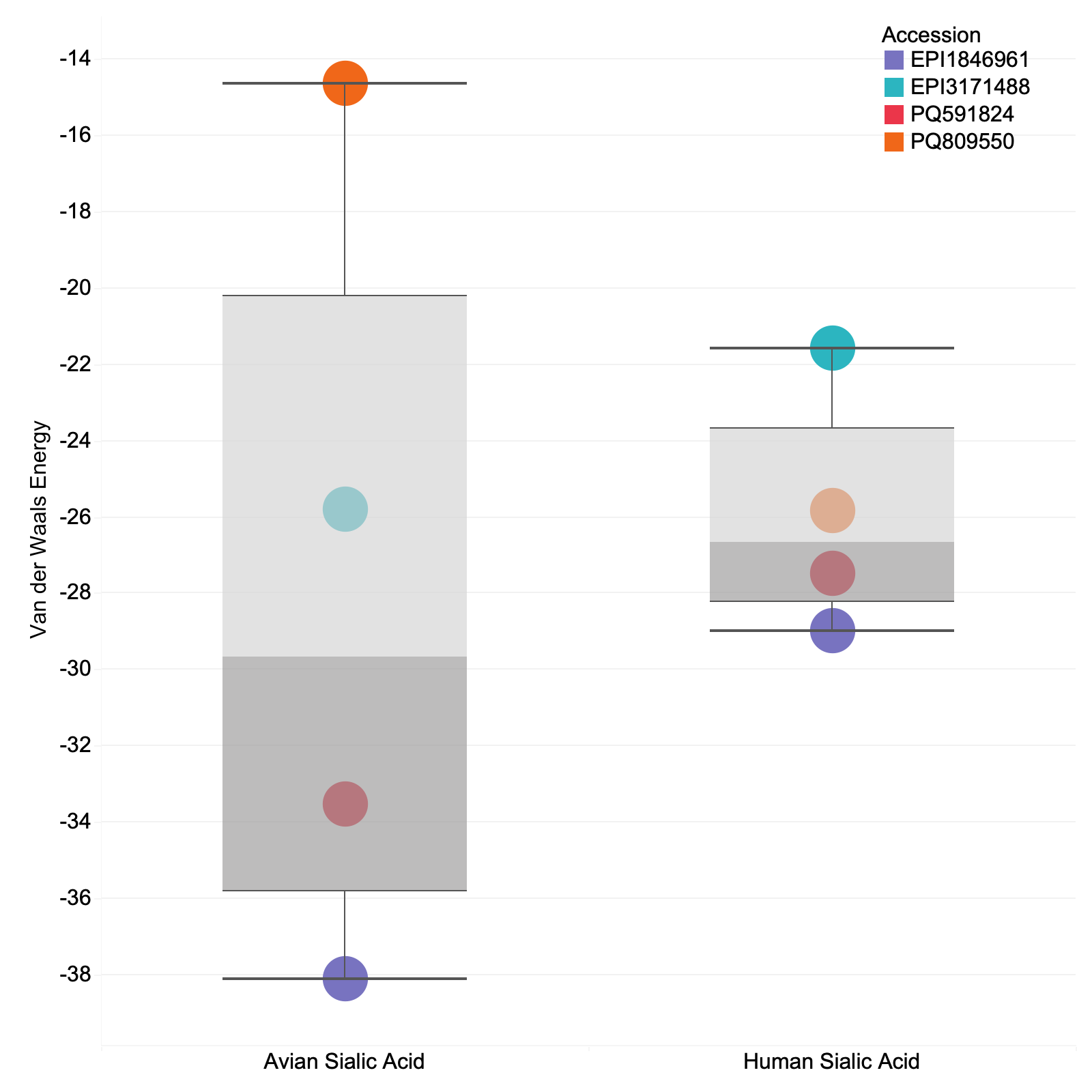

Supplement: Supplementary 1 — Files S1 to S23 Figs. S24 to S30 [file csbj.0066.f1.zip › supplemental_fig_28.png]

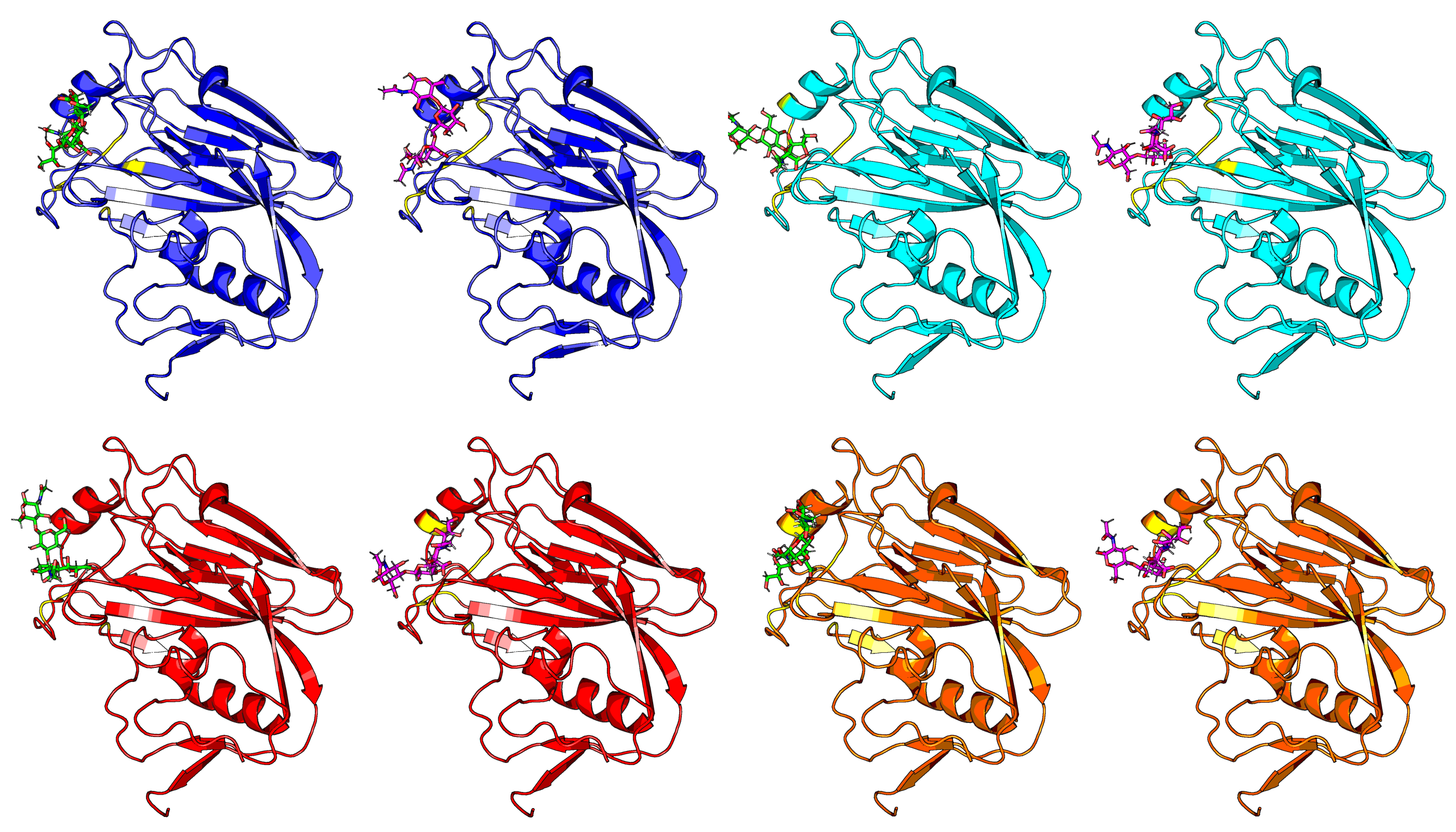

Supplement: Supplementary 1 — Files S1 to S23 Figs. S24 to S30 [file csbj.0066.f1.zip › supplemental_fig_29.png]

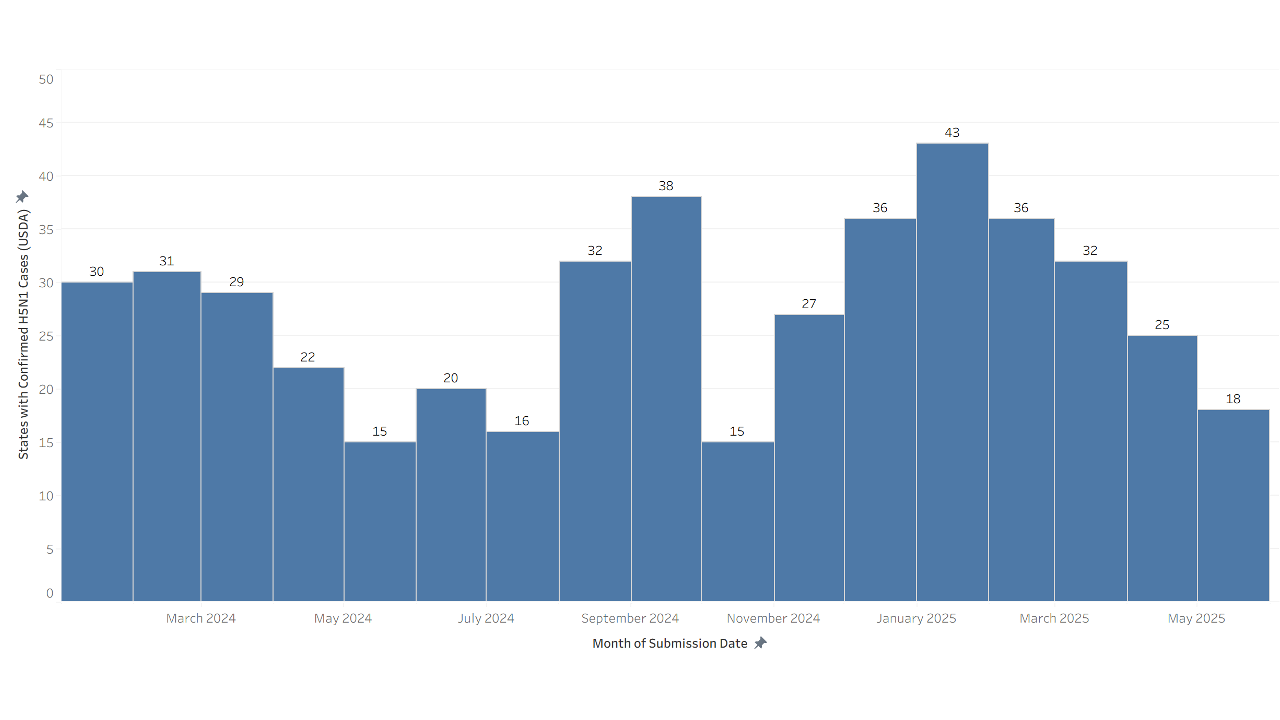

Supplement: Supplementary 1 — Files S1 to S23 Figs. S24 to S30 [file csbj.0066.f1.zip › supplemental_fig_30.png]
